# The influence of altitude and landforms on some biochemical and hematological parameters in Ouled Djellal ewes from arid area of South East Algeria

**DOI:** 10.14202/vetworld.2015.130-134

**Published:** 2015-01-31

**Authors:** Mohammed Titaouine, Toufik Meziane

**Affiliations:** 1 Department of Veterinary Medicine, Laboratory of Environment, Animal Health and Production, University of El-Hadj Lakhdar, Batna 05000, Algeria; 2 Department of Nature and Life Science, University of Mohamed Kheider, Biskra 07000, Algeria

**Keywords:** altitude, arid area, biochemistry, hematology, landform, Ouled Djallel ewes

## Abstract

**Aim:**

This study was conducted on Ouled Djellal ewes in arid area of south-east Algeria in order to reveal the influence of altitude and landforms on some hematological and biochemical parameters.

**Materials and Methods:**

A total of 160 ewes having 3-5 years of age, multiparous, non-pregnant, non-lactating and reared in arid areas of South East Algeria were included. Blood samples were divided according to factors of altitude and landform (plain region at 150 m above sea level, tableland region at 600 m above sea level and mountain region at 1000 m above sea level). The whole blood was analyzed for hematology, and plasma samples for biochemical analysis.

**Results:**

The study found lowest glucose concentrations were detected in tableland region at 600 m. In plain region at 150 m, ewes had a higher (p<0.01) concentration of cholesterol and triglyceride. Furthermore, a higher concentration of total proteins (p<0.01) and urea (p<0.05) were detected in plain region at 150 m. The average blood creatinine concentration in mountain ewes at 1000 m and tableland ewes at 600 m were higher (p<0.05) that in plain ewes at 150 m. The highest calcium concentration was found at the altitude of 150 m and the lowest at the altitude of 1000 m (1.12±0.35 mmol/L vs. 0.52±0.03 mmol/L). Phosphorus levels were higher at altitudes of 150 m than at the altitude of 600 m and 1000 m (0.93±0.42 mmol/L vs. 0.68±0.54 mmol/L, 0.23±0.01 mmol/L). The highest hemoglobin concentration and value of hematocrit were detected in mountain ewes at the altitude of 1000 m (120.61 g/L, 40%) and the lowest at the altitude of 150 m (73.2 g/L, 31%) (p<0.001).

**Conclusion:**

We concluded that hematological and biochemical parameters in Ouled Djellel ewes reared in arid area may be affected by altitude and landforms.

## Introduction

Sheep breeding constitutes an important source of animal protein. However, productivity varies considerably from one region to another depending on the breeds, breeding systems, methods of herding and physical environment. In Algeria, the sheep herds are distributed mainly in the steppe exploiting the desert pastures, traditionally managed in almost all farms. Several breeds constitute the Algerian sheep population whose Ouled Djellal breed that is numerically the largest national herd 50% [1], this breed recognizes that a good quality breeding and adaptation to harsh environmental conditions [2,3]. Understanding the mechanisms of response and organism adaptation of these animals face environmental challenges of arid regions (ambient temperature, relative humidity, altitude, solar radiation and wind speed…) is essential for the implementation of improvement programs to reduce the adverse impacts of climate change [4,5]. Thus, the main objective of this study is to determine the values of several biochemical and hematological parameters in Ouled Djellal ewes in arid conditions and to analyze the influence of altitude with landforms on those values.

## Materials and Methods

### Ethical approval

Animal experiment was conducted in herds reared under extensive condition. All adequate measures were taken to erase pain and discomfort in accordance with the International Animal Ethics Committee. The permission to collect the samples from live sheeps was accorded by the committee framed for the research by the university authority.

### Study area

The investigations were conducted in farms located in the arid region of South Eastern Algeria. The study area is characterized by large diurnal temperature variations. Thus, the temperature which often reaches 48-49°C in the shade during the months of July and August in the day, down to around 15°C at night. The change in daily temperature range is important during all months of the year. The maximum temperature is reached in the month of July 49°C, against in January it hovers around 5°C (especially at night). Precipitation is not important. Seasonal variability is marked by the concentration of rainfall in winter 37.5% and spring 30.7%, the rest of the rain as the storm during summer and fall. The rainfall deficit peaked in the months of July and August. For evaporation, the region is characterized by a very high evaporation exceeding 2300 mm/year resulting from a high temperature, low humidity and prevailing wind. Altitude, landforms, rainfall and temperature for the three study areas are shown in Table-1.

**Table-1:** The main characteristics of the study areas.

	Region 1	Region 2	Region 3
Altitude (m)	150	600	1000
Landforms	Plain	Tableland	Mountain
Rainfall mm/month	10.4	12.85	12.02
Annual temperature°C			
Max	32.1	26.9	23.2
Min	20.3	15.2	12.4
Mean	26.2	21.05	17.8

### Animals and management

The study included 160 Ouled Djellal ewes, excellent meat breed, adapted to arid areas, dominant in the region, multiparous, 3-5 years, non-pregnant, non-lactating and having a note average of body condition score 2.25±0.5, which were divided into three lots in three regions according to altitude and landform:

Region 1 (Group I): Ewes were managed in farm located in plain area at 150 m above sea level (n=60).

Region 2 (Group II): Placed in the tableland region, the farm located at 600 m above sea level (n=60).

Region 3 (Group III): The animals reared on the mountain region at elevation 1000 m above sea level (n=40).

Animals were fed only on natural pasture, which contains plant species of the steppe, including *Stipa tenacissima*, *Ampelodesmos tenax* and by annual grasslands, composed of various grasses (*Cynodon dactylon* predominantly) and legumes (*Melilotus sulcata* and *Vicia monantha*), and drink natural water. Regions are far away from all kinds of urbanization and industrialization.

All ewes are routinely vaccinated for sheep pox, peste des petits ruminants, enterotoxaemia and administered anthelmentic prophylactics 4 times/year.

### Blood samples

To facilitate the restraint and limit variations related to food intake and stress, the blood samples were taken by puncture of the jugular vein in the early morning before food intake. 7 ml of blood was collected using sterilized needles and plastic syringe from external jugular vein in tubes with heparin anticoagulant. Blood samples were divided into two aliquots. One aliquot was used for estimation of hemoglobin (Hb) concentration and hematocrit (packed cell volume) while the other was used for plasma separation. Plasma was separated from the blood by centrifugation at 3500 rpm at room temperature for 20 min. The plasma was divided into aliquots in microcentrifuge tubes and kept frozen at −20°C till further analysis. Plasma samples were used to estimate biochemical parameters.

### Analytical methods

The biochemical parameters; plasma glucose, plasma triglyceride, total plasma cholesterol, total plasma protein, uremia, total plasma creatinine, plasma calcium and plasma phosphorus, were estimated using commercial kits “SPINREACT,” Spain as per standard method using the ultra violet (UV)-visible recording spectrophotometer (UV-160A; Shimadzu Corporation, Japan). The hematological parameters were determined as follows: Hb was determined by the method to the cyan-met-Hb described by Van Kampen and Zijlstra [6]. The hematocrit value was determined by the Janetzki capillary microhematocrit method.

### Statistical analysis

Statistical analysis was carried out by Student’s t-test to compare the means of two groups by using the Minitab version 15. The significant difference at p<0.05 was considered.

## Results

Altitude and landforms influence on some biochemical and hematological parameters in Ouled Djellal ewes from arid area of South East Algeria are set out in Tables-2 and 3. Mean plasma glucose of ewes in plain region at 150 m differed significantly (p<0.01) only with ewes in tableland region at 600 m. However, plasma glucose of ewes at 150 m did not differ significantly from the mountain ewes at 1000 m (region 3). In addition, plasma glucose of ewes in tableland region differed significantly from the mountain ewes, the serum cholesterol and triglyceride levels recorded in mountain region and tableland region were significantly lower than in plain region (p<0.01).

**Table-2:** Concentrations of biochemical parameters measured on 160 Ouled Djellal ewes of South East Algeria (arid area) depending on the altitude and landform.

Parameters	Region 1 Group I (M±SD)	Region 2 Group II (M±SD)	Region 3 Group III (M±SD)	p value		

				Group I versus Group II	Group I versus Group III	Group II versus Group III
Glucose (mmol/L)	3.77±1.22	1.83±1.72	3.82±0.33	<0.01	NS	<0.05
Cholesterol (mmol/L)	1.29±0.75	1.24±0.75	0.93±0.31	<0.01	<0.001	NS
Triglycerides (g/L)	0.25±0.01	0.22±0.16	0.22±0.07	<0.01	<0.01	NS
Total protein (g/L)	68.14±15.89	65.91±19.87	59.13±23.5	<0.05	<0.01	NS
Urea (mmol/L)	9.63±3.57	8.56±1.78	7.14±2.49	<0.05	<0.05	NS
Creatinine (µmol/L)	105.81±18.74	119.16±36.77	119.60±35.71	<0.05	<0.05	NS
Calcium (mmol/L)	1.12±0.35	1.01±0.58	0.52±0.03	NS	<0.01	<0.05
Phosphorus (mmol/L)	0.93±0.42	0.68±0.54	0.23±0.01	NS	<0.01	<0.01

Results are expressed as mean±standard deviation. NS=Non-significant

**Table-3:** Concentrations of hematological parameters measured on 160 Ouled Djellal ewes of South East Algeria (arid area) depending on the altitude and landforms.

Parameters	Région 1 Group I (M±SD)	Région 2 Group II (M±SD)	Région 3 Group III (M±SD)	p value		

				Group I versus Group II	Group I versus Group III	Group II versus Group III
Hb (g/L)	73.2±25.4	86.47±38.5	120.61±33	NS	<0.01	NS
Hématocrite (%)	31±4	36±5	40±1	NS	<0.001	<0.01

Results are expressed as mean±standard deviation. NS=Non-significant, Hb=Hemoglobin

Total protein and plasma urea levels were significantly higher in plain region at 150 m than in tableland region at 600 m (p<0.05) and mountain region at 1000 m (p<0.01). The plasma creatinine concentrations were significantly higher in region 3 and in region 2 compared with in region 1 (p<0.05).

Plasma calcium and phosphorus levels in region 3 were significantly lower than those in region 1 and region 2 (p<0.01).

Moreover, altitude and landform affected some hematological parameters (Table-3), Thus the rate of Hb was the lowest recorded in plain region at 150 m (73.2±25.4g/L) and the highest rates in mountain region at 1000 m (120.61±33 g/L) (p<0.01) as well, hematocrit of mountain ewes at 1000 m altitude is significantly higher than those in other regions (p<0.001 for region 1 and p<0.01 for region 2).

## Discussion

In our study, plain region (150 m above sea level) is hotter than the tableland region (600 m above sea level), plasma glucose increased significantly in plain ewes as compared to tableland ewes because the insulin levels decrease in animals exposed to high temperature [7,8]. On the other hand, it is assumed that this is also probably due to the increased glucose demand due to increased respiratory muscular activity after thermal exposure [9].

Glycaemia of ewes in the region 3 is higher than that of other regions, indeed, of such variation can reported to the importance of physical activity; because the region 3 at 1000 m above sea level, is a mountainous region and these animals are more energetic and combative that animals of other regions; the same results found by Gustafson *et al*. [10] in cows under the effect of physical efforts. However, other authors [11,12] showed a decrease in blood glucose following a physical activity.

The overall mean of blood cholesterol decreases with altitude. These results are similar to those of El-Masry and Marai [13] on dairy cattle and Okab *et al*. [14] on rabbits. They attributed these changes to variations in thyroidal activity at different climatic conditions; in tableland and mountain regions, the exposure to low environmental temperature compared with that of plain region stimulates the secretion of thyroxin. Thyroid hormones stimulate cholesterol synthesis as well as the hepatic mechanisms that remove cholesterol from the circulation. The decline in plasma cholesterol level because the rate of the later process exceeds that of the former.

Total protein appeared in this study, higher in plain region at 150 m than in other regions. The importance of increasing total protein in plain ewes, which suffer more from heat, may be due to the fact that total protein in plasma generates a colloid osmotic pressure which controls the flow of water between blood and tissue fluids [15].

Concerning uremia, this parameter was influenced by altitude in Ouled Djellal ewes, such as total protein, urea seems playing an important role in the dehydration of the animals following an increase in environmental temperature. Indeed, by its osmotic effects, urea serves to draw water from other areas to the plasma. Tubular reabsorption of urea is under hormonal influence (antidiuretic hormone). In fact, the active reabsorption of water is accompanied by that of urea. In our study, creatinine appeared lower in Group I ewes (at 150 m), creatinine is formed by irreversible dehydration of creatine phosphate in muscle, and it is increased by the content of creatine in the body, which is directly related to muscle mass and therefore the average body condition of the animal; is associated with muscular dystrophy or exercise [16]; Therefore, high levels of creatinine in the mountain ewes (at 1000 m), which have considerable physical activity during their displacement by contribution of other regions. Similarly, other authors [8,17,18] reported that the major exogenous regulator of thyroid gland activity is environmental temperature, the exposure of animals to high ambient temperature was associated with depression of thyroid activity thereby causing a relatively lower concentration of thyroid hormones [8], in the present study the temperature difference between three areas, supposes increased thyroid activity in mountain ewes (Group III), causing an amplification of muscle protein catabolism and increased creatinine production. The significant decrease in plasma calcium and phosphorus in mountain ewes in this study could be to excessive demand for muscular activity of these animals; more Srikandakumar *et al*. [19] demonstrated that the total plasma calcium is affected by total plasma protein concentration as approximately 45-50% of the total plasma calcium is bound to plasma proteins. Accordingly, plasma calcium concentration will be decreased with hypoproteinemia. The highest Hb concentration in Ouled Djellel ewe blood was recorded in mountain region at 1000 m. The effect of altitude on erythrocytic values has been studied by many investigators. Soch *et al*. [20,21] demonstrate that the reduction of oxygen pressure in highland regions leads to an increased production and release of erythropoietin, thereby stimulating erythropoiesis as a coping or adaptive mechanism to low oxygen levels in such environments. Therefore, the higher Hb and hematocrit values of Ouled Djellal ewes at the altitude of 1000 m a.s.l. could provide evidence of the adaptation of these breed to low atmospheric oxygen [22]. In addition, Hb and hematocrit are considered to be the indices of the organic response to exercise [23,24]. The increased Hb and hematocrit in mountain ewes could result in increasing the oxygen-carrying capacity of the blood to support the severe muscular activity in these ewes.

## Conclusion

The altitude with landform are likely to induce variations in the concentrations of various biochemical and hematological parameters in Ouled Djellal ewes reared under extensive condition, although the study was conducted in arid areas of south eastern Algeria, which is relatively rare, altitude and landform significantly affects plasma glucose, triglyceride, cholesterol, total proteins, blood urea, plasma creatinine, plasma calcium, phosphorus, Hb and hematocrit. However, further studies are required to complete this study, including the identification of endocrine parameters, micro-minerals, other hematological parameters, and more research on the effects of age, sex, season and the muscular exercise to discover a clearer picture of altitude impact on growth and reproduction and, accordingly, the counter-measures needed to be developed. This will ensure a sizeable improvement in the economy of sheep farms by minimizing the production loss in the arid area of Algeria.

## Recommendations

The study establishes the adaptive capability of Oulled Djellel ewes to the different altitude and landforms in the arid environment. As, generally, livestock reared in hot arid environments need to displace long distances for grazing, our finding has a greater significance in terms of improving the performance of these animals in such environmental extremes. The study gives a clue that animals at deferent altitude and landform need to be supplemented with a higher energy feed compensate for the physiques activity and water supplementation to counter the high temperature as well as to maintain production.

## Authors’ Contributions

TM performed blood sampling of animals and determination of blood parameters in the laboratory. TM and MT drafted and revised the manuscript. All authors read and approved the final version of the manuscript.
